# Saliva-based SARS-CoV-2 serology using at-home collection kits returned via mail

**DOI:** 10.1038/s41598-022-17057-7

**Published:** 2022-08-18

**Authors:** Christopher Campbell, Douglas Roblin, Nikhil Padmanabhan, Daniel Romero, Jessica Joe, Lily Fathi, Thomas Whiting, Jared Williamson, Paul Goodwin, Charmaine Mckie, Adrienne Deneal, Leslie Greenberg, George Sigal

**Affiliations:** 1 Meso Scale Diagnostics, LLC., Rockville, MD USA; 2 Mid-Atlantic Permanente Research Institute, Kaiser Permanente, Rockville, MD USA; 3 Meso Scale Diagnostics, LLC, 16020 Industrial Drive, Gaithersburg, MD 20877 USA

**Keywords:** Antibodies, Biomarkers

## Abstract

Serology provides tools for epidemiologic studies, and may have a role in vaccine prioritization and selection. Automated serologic testing of saliva, especially specimens that are self-collected at home and sent to a laboratory via the mail without refrigeration, could be a highly-scalable strategy for population-wide testing. In this prospective study, non-vaccinated patients were recruited after PCR testing to self-collect saliva and return their specimens via mail. Longitudinal specimens were analyzed in order to monitor seroconversion in the weeks after a diagnostic PCR test for SARS-CoV-2. Diverse users self-collected saliva and returned specimens via mail in compliance with shipping regulations. At our pre-established threshold (0.963 AU/mL), salivary IgG reactivity to full-length spike protein achieved 95.8% sensitivity and 92.4% specificity at 2–4 weeks after diagnostic testing, which is comparable to the typical sensitivity and specificity achieved for serum testing. Reactivity to N antigen also was detected with 92.6% sensitivity and 90.7% specificity at 4–8 weeks after diagnostic testing. Moreover, serologic testing for endemic coronaviruses performed in multiplex with SARS-CoV-2 antigens has the potential to identify samples that may require retesting due to effects of pre-analytical factors. The easy-to-use saliva collection kit, coupled with thresholds for positivity and methods of flagging samples for retest, provides a framework for large-scale serosurveillance of SARS-CoV-2.

## Introduction

Serology assays measuring the development of host antibodies against SARS-CoV-2 antigens are important tools in the public health response to COVID-19. At the population level, serology can be used for disease surveillance and the evaluation of public health countermeasures to decrease transmission rates, and is a critical component of clinical studies to qualify new vaccines^1–4^. Serosurveillance can be invaluable for determining the case fatality rate, tracking incidence and prevalence, and investigating medical sequelae following recovery^5,6^. Serology has also been used in population studies to evaluate approaches to vaccine prioritization^7^ and dosing^8^ and to assess the durability of antibody production as a correlate of immunity after vaccination^9^ or natural infection^10^. At the individual level, there is some debate about the utility of serology assays for diagnosing acute COVID-19 infection^11^; however, the assays provide a tool for diagnosing post-acute or chronic COVID-19 symptoms in individuals who may have had asymptomatic or undiagnosed acute infections^12^. Serology assays are also likely to become increasingly important, as the correlation of antibody levels and protection from infection becomes better defined, for identifying individuals in need of vaccine boosters^1,3^.

The use of serology assays in some applications, especially for serosurveillance, is limited by the use of serum as the most common sample matrix and the need to collect blood from the target population. To take advantage of blood that is being collected for other purposes, many serosurveillance studies have relied on testing of blood at dialysis or blood donation centers^13–17^. Expanding serosurveillance to a diverse population presents several logistical challenges to obtain, process, and transfer serum. Self-collected specimens reduce some of these logistical challenges^18–21^. Saliva is especially easy to self-collect without pain or the use of needles or lancets. The bulk of salivary IgG is thought to originate from blood due to transudation or bleeding from the gingival tissue^22–24^. The potential of saliva as a sample for SARS-CoV-2 serosurveillance is supported by the strong correlation that has been observed in the antibody response in serum and saliva during acute infection and recovery^25–29^. Although the FDA has cleared serology tests for HIV^30,31^, saliva-based serology tests for SARS-CoV-2 are not yet commercially available.

Considerations in developing a saliva-based serology test include ease of use, regulatory requirements, and demands of downstream processing. A prior study found that patients can confidently and correctly self-collect specimens at home^19^. Other prior studies^29,32^ supervised saliva collection in research settings using devices that have not been FDA approved and/or need secondary shipping containers to meet UN3373 category B requirements. Numerous methods and devices have been designed to collect oral fluids^33,34^. Some use swabs or sponges to collect saliva from under the tongue, or the fluid at the interface of teeth and gums (gingival crevicular fluid). Swab-based methods are especially convenient for young children and people who find handling or viewing liquid saliva unpleasant. Extraction of saliva from swabs, however, requires additional processing steps. A FDA registered device from Salimetrics is commercially available that enables a clean and simple protocol to collect liquid saliva into a tube by drooling through a fit-for-purpose straw^34^.

Previously, we developed and pilot tested an at-home saliva-collection kit using the Salimetrics device^35^. Specimens were returned via mail without refrigeration or preservatives. Moreover, streamlined processing techniques were developed for assaying saliva using quantitative, multiplexed immunoassays that can be automated to achieve the high throughput required for population-level testing.

Here, we evaluated the sensitivity and specificity of a “spit and mail” serology test using prospective samples and pre-established thresholds set by our pilot study. By testing samples provided from non-vaccinated individuals in the weeks following nasal PCR testing for SARS-CoV-2, our study addressed questions relevant for performing a large-scale serological survey. We verified the ability of a diverse group of participants to return specimens in compliance with shipping regulations using only written instructions without supervision or in-person training. We measured the dynamics of salivary anti-CoV-2 antibodies and computed the sensitivity and specificity at various time points after diagnostic PCR testing. Additionally, we compared strategies of identifying samples that may have been affected by pre-analytical issues inherent in shipping self-collected saliva without preservatives or refrigeration.

## Results

### Received specimens

We received self-collected saliva from 121 unique participants: 40 participants (33%) who tested positive for SARS-CoV-2 infection by RT-PCR and 81 participants who tested negative (67%). As shown in Table 1, the participants were diverse in age, gender, ethnicity, and socioeconomic status (SES). The samples were collected from non-vaccinated individuals from July 2020 to March 2021 within the Washington, DC metropolitan area, prior to the broad availability of SARS-CoV-2 vaccines. Participants who tested positive had similar demographics to participants who tested negative. The groups were well-balanced by gender and SES, as indicated by the area disadvantage index (ADI)^36^. Participants whose PCR test did not detect SARS-CoV-2 (PCR- participants) tended to be slightly older and more likely to be insured through Medicare than participants whose PCR test detected SARS-CoV-2 (PCR + participants). This trend may result from a lower positivity rate among older asymptomatic patients undergoing pre-surgical clearance for outpatient surgery than among patients tested due to symptoms of COVID-19. Additionally, PCR + participants were less likely to identify as non-Hispanic White, reflecting the rates of COVID-19 infection among different ethnic groups^37^.

**Table 1:** Demographics of study participants.

	Number of participants	Percentage of all participants	Result of SARS-CoV-2 PCR test at recruitment				P-value for difference between SARS-CoV-2 PCR test result (detected vs. not detected)
			Detected (N = 40)		Not detected (N = 81)		
			Number of PCR + participants	Percentage of all PCR + participants	Number of PCR − participants	Percentage of all PCR − participants	
**Age**							
≥ 75 years	7	5.79	1	2.50	6	7.41	0.31
65–74 years	23	19.01	5	12.50	18	22.22	
50–64 years	42	34.71	15	37.50	27	33.33	
18–49 years	49	40.50	19	47.50	30	37.04	
**Gender**							
Female	63	52.07	21	52.50	42	51.85	0.95
Male	58	47.93	19	47.50	39	48.15	
**Race/ethnicity**							
Hispanic	3	2.48	1	2.50	2	2.47	0.07
Non-Hispanic Black	40	33.06	16	40.00	24	29.63	
Non-Hispanic White	73	60.33	19	47.50	54	66.67	
Other/unknown	5	4.13	4	10.00	1	1.23	
**Health plan**							
Standard HMO	71	58.68	29	72.50	42	51.85	0.07
High deductible	8	6.61	2	5.00	6	7.41	
Medicare	31	25.62	6	15.00	25	30.86	
Medicaid	4	3.31	0	0	4	4.94	
Other/unknown	7	5.79	3	7.50	4	4.94	
**Area disadvantage index**							
Highest SES qtl	47	38.84	14	35	33	40.74	0.85
Upper mid-qtl	42	34.71	15	37.5	27	33.33	
Lower mid-qtl	15	12.4	6	15	9	11.11	
Lowest SES qtl	17	14.05	5	12.5	12	14.81	

p-values were computed using the chi-square test. Fisher’s exact tests were also run in consideration of small cell sizes. In all cases, the p-values for the chi-square or Fisher’s tests were > 0.05.

Participants were instructed to return specimens immediately upon receiving an enrollment kit and then again at 10 and 30 days after their PCR test. Among participants who provided a specimen, the majority provided three specimens as instructed; however, the timing of collection and mailing in some cases varied considerably from the specified times. Samples were mailed by participants as early as 1 day and as late as 102 days after their RT-PCR test. The mode and median of days between PCR test and mailing the first sample were 9 and 11 days, respectively. To create groups with roughly equal numbers of samples, samples were divided into three time categories depending on whether they were mailed by participants < 2 weeks, 2–4 weeks, or 4–8 weeks after the PCR test (Table 2).

**Table 2:** Overview of received specimens.

	Result of SARS-CoV-2 PCR test at recruitment			
	Not detected (n = 81)		Detected (N = 40)	
	Number of PCR − participants	Percentage of all PCR − participants	Number of PCR + participants	Percentage of All PCR + participants
**Time between PCR test and when sample mailed**				
≤ 2 weeks	47	58.0%	28	70.0%
2–4 weeks	47	58.0%	25	62.5%
4–8 weeks	63	77.8%	27	67.5%
> 8 weeks	15	18.5%	2	5.0%
**Number of specimens returned by participant**				
1 Specimen	14	17.3%	7	17.5%
2 Specimens	10	12.3%	9	22.5%
3 Specimens	57	70.4%	24	60.0%

Table shows number of participants who returned a specimen within the indicated time after a PCR test for SARS-CoV-2. Additionally, numbers of participants returning the indicated number of specimens are listed.

The transit time of specimens from participants to the laboratory ranged from 1 to 31 days (1st quartile = 1.4 days, median = 1.9 days, 3rd quartile = 3.5 days) in the mail with 91% arriving in less than 5 days, which was our target window based on prior testing showing stability for 5 days^35^. Two specimens that were in the mail over 20 days were excluded from analysis of test performance, but were included in an analysis investigating potential indicators of sample degradation.

### IgG reactivity to SARS-CoV-2 antigens

**Figure 1:**
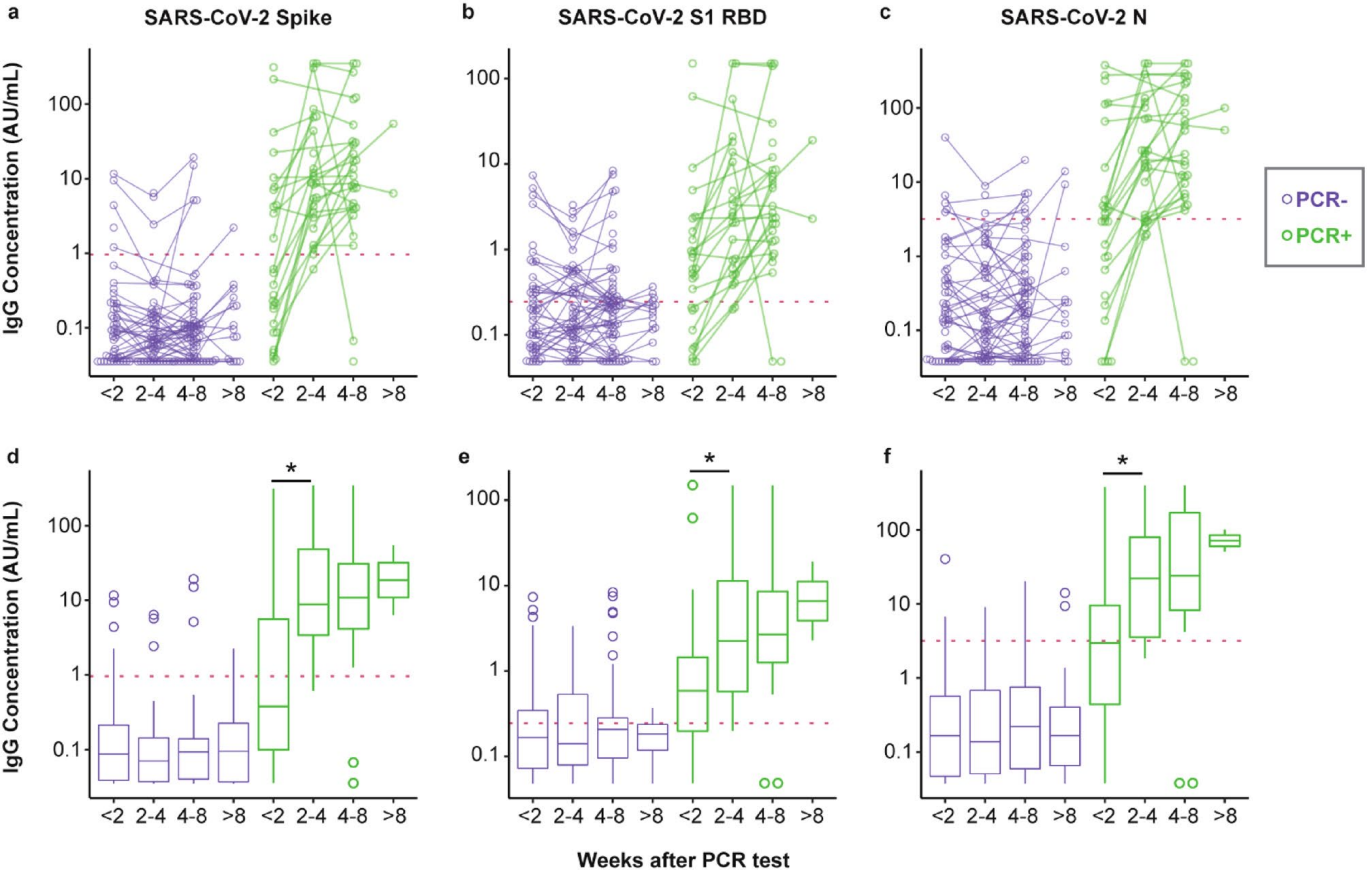
Salivary anti-SARS-CoV-2 IgG antibodies in the weeks after a PCR test. Reactivity to (**a**) full-length spike protein, (**b**) receptor binding domain (RBD) of the spike protein, and (**c**) N protein of SARS-CoV-2 were measured in saliva provided by 121 participants who had received a PCR test for SARS-CoV-2. Participants provided up to three samples. Matched samples provided by the same donor are connected with lines. 81 participants tested negative for SARS-CoV-2 (colored purple), and 40 participants tested positive (colored green). The dashed, red line indicates the pre-established cut-point for high versus low antibody levels. (**d**–**f**) Box and whisker plots of the same data shown in panels (**a**–**c**). The PCR + cohort was significantly different (p < 0.001; see Supplemental Tables) from the PCR- cohort at all timepoints. Asterisks indicate statistical significance (p < 0.05 by Mann–Whitney test) for difference between the < 2 weeks and 2–4 weeks timepoints.

Concentrations of salivary IgG reactive to coronavirus antigens are shown in Fig. 1. Antibody positivity for SARS-CoV-2 antigens was determined based on pre-established thresholds set at the 98th percentile for saliva self-collected from presumed naive participants (no PCR confirmed diagnosis, no household exposure, and no symptoms of COVID-19) in a previous study^35^. Pre-established IgG thresholds for Spike, RBD, and N were 0.963, 0.244, and 3.18 AU/mL, respectively.

Clinical performance of the serology assays was determined relative to the COVID-19 PCR test result at enrollment. Sensitivity and specificity were calculated, respectively, as (i) the proportion of saliva specimens from PCR-confirmed cases with antibody levels above the pre-established thresholds and (ii) the proportion of saliva specimens from PCR-negative cases with antibody levels at or below the pre-established thresholds. Measured sensitivity and specificity values are provided in Table 3. The SARS-CoV-2 Spike IgG assay provided the best overall accuracy. The sensitivity was only 40.7% within two weeks of PCR testing, but increased to 96.0% at 2–4 weeks and 92.6% at 4–8 weeks after PCR testing. The specificity was 92.4%. By comparison, when the same assay was evaluated with serum samples in an independent study, the sensitivity and specificity were reported as 90.8% and 97.4%, respectively^38^. The SARS-CoV-2 N IgG assay performed similarly to the SARS-CoV-2 Spike assay with point estimates for sensitivity and specificity that were not statistically different. The SARS-CoV-2 RBD IgG assay exhibited similar sensitivity; however, the specificity was significantly poorer (Table 3), which may indicate that the pre-set assay threshold was not optimal (see discussion of threshold verification below).

**Table 3:** Sensitivity and specificity of salivary IgG for detection of prior SARS-CoV-2 infection.

Antigen	Isotype	Threshold (AU/mL)	Sensitivity at < 2 weeks	Sensitivity at 2–4 weeks	Sensitivity at 4–8 weeks	Specificity
SARS-CoV-2 Spike	IgG	0.963	40.7% (22.4–61.2%)	95.8% (78.9–99.9%)	92.6% (75.7–99.1%)	92.4% (87.4–95.9%)
SARS-CoV-2 N	IgG	3.18	48.1% (28.7–68.1%)	79.2% (57.8–92.9%)	92.6% (75.7–99.1%)	90.7% (85.3–94.6%)
SARS-CoV-2 S1 RBD	IgG	0.244	66.7% (46.0–83.5%)	91.7% (73.0–99.0%)	92.6% (75.7–99.1%)	64.5% (56.9–71.7%)

Point estimates and 95% confidence intervals (indicated in parentheses) were computed at pre-established thresholds.

**Figure 2:**
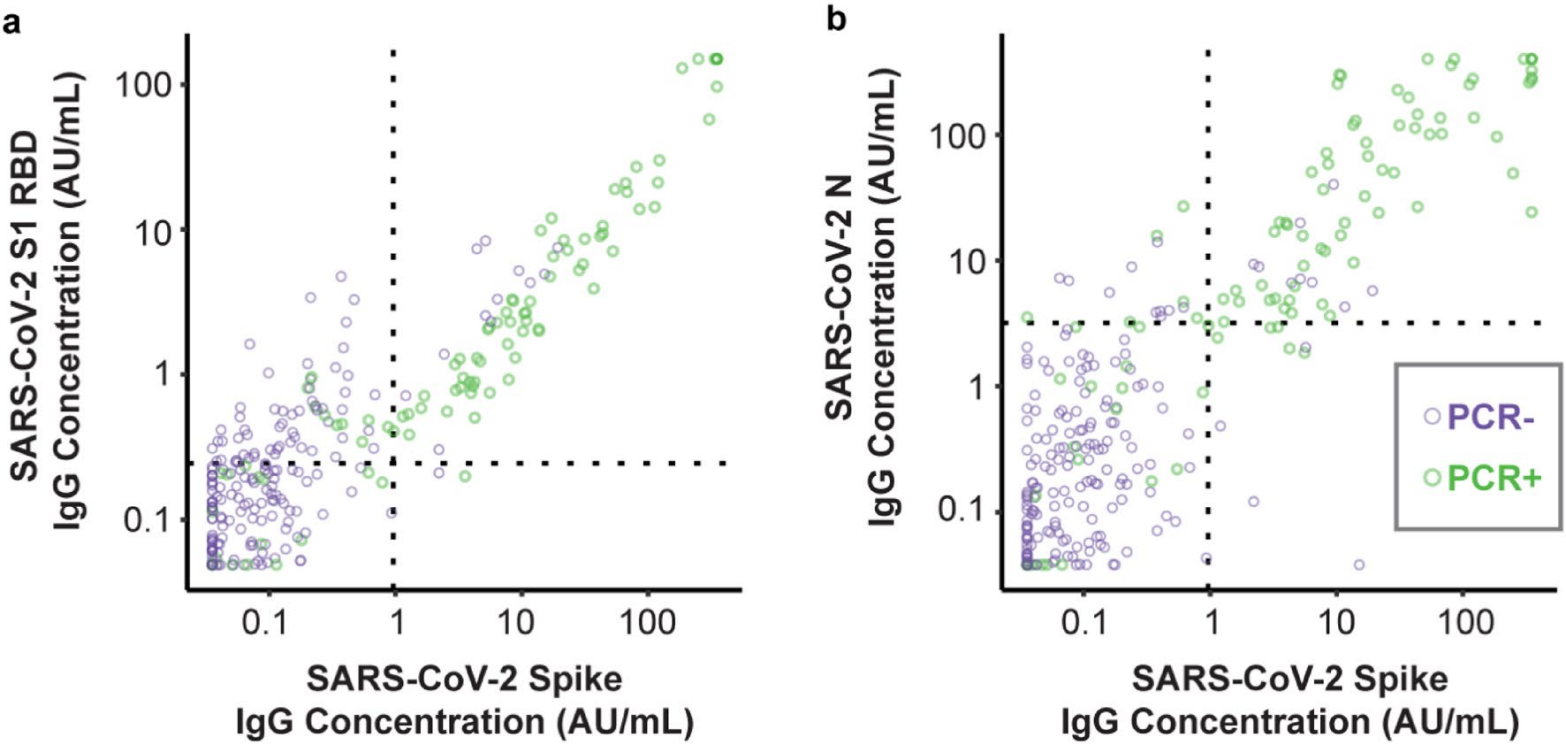
Correlation in IgG concentrations for SARS-CoV-2 antigens. Dotted lines indicate thresholds for classifying high versus low reactivity.

IgG reactivity to the SARS-CoV-2 spike protein was highly correlated with IgG reactivity to the N protein and RBD domain of spike protein, especially for samples from PCR-positive cases (Fig. 2). The correlation of the reactivities to RBD and Spike (Fig. 2a) shows that the concentrations of anti-RBD IgG antibodies tended to be about threefold lower than for full-length spike. As RBD is a fragment of the Spike protein, the difference in antibody activity is likely due to the reduced number of antigenic epitopes displayed for RBD relative to Spike. For PCR-negative individuals, measured reactivities of IgG to SARS-CoV-2 N tended to span a larger range than IgG to SARS-CoV-2 Spike, which was heavily skewed to the bottom of the assay range. This result may be a consequence of cross-reactive host antibodies from previous infections with other circulating coronaviruses, since the N protein has greater conservation across human coronaviruses than the Spike protein. However, the effect of any cross-reactivity on assay performance was small, with the N assay showing only a small and non-statistically significant decrease in specificity relative to the Spike assay.

We looked for evidence that elevated antibody levels in PCR-negative participants may have been due to undiagnosed infections. For the 67 PCR-negative participants who provided at least two samples, 6 of these 67 participants had at least one sample above the assay threshold for the SARS-CoV-2 Spike IgG assay. Of these 6 participants with at least one positive sample, 2 had salivary IgG levels above the threshold for SARS-CoV-2 Spike for all three of their samples. These participants also had salivary IgG levels above the threshold for SARS-CoV-2 N protein, which suggests an undiagnosed infection prior to enrollment. Of the 6 participants with at least one positive sample, 2 participants had an initial negative sample and then showed delayed sero-conversion after 30 days. The sero-conversion was also observed using the SARS-CoV-2 N IgG assay, which suggests that these participants may have been infected at enrollment and received a false negative PCR test^39^, or they may have become infected after enrollment.

### Verification of pre-established thresholds

**Figure 3:**
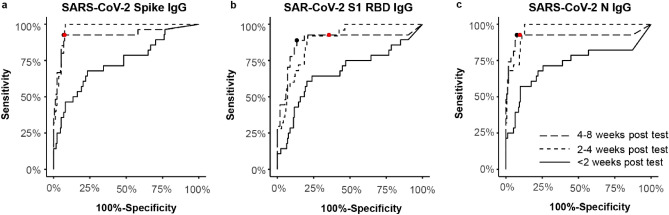
Receiver-operator characteristic (ROC) curves for classifying SARS-CoV-2 PCR results based on salivary anti-SARS-CoV-2 IgG antibodies. Curves are drawn to show performance of assay at < 2 weeks, 2–4 weeks, and 4–8 weeks after a PCR test. The red dot indicates sensitivity and specificity achieved at the 4–8 week time point for the pre-established threshold. The black dot indicates, on the same curve, the threshold that maximizes sum of sensitivity and specificity.

Receiver operator characteristic (ROC) curves were generated (Fig. 3), and the area under curve (AUC) values for the ROC curves were calculated (Supplemental Table 1) to compare the diagnostic performance of the serology assays at different times after nasal PCR testing and to confirm that the pre-determined thresholds were optimal for identifying infections. For all three SARS-CoV-2 antigens, the area under the curve (AUC) was significantly greater for samples collected more than two weeks after PCR testing relative to samples collected within 2 weeks of testing, largely reflecting the higher sensitivity that was observed for the later samples (Table 3). ROC curves for samples and the associated AUC values were not significantly different for samples collected 2–4 weeks and > 4 weeks after PCR testing, indicating that the assay achieved optimal diagnostic performance by the 2 week time point. The ROC curves for the SARS-CoV-2 Spike and N IgG assays were similar with AUC values of 0.926 and 0.916, respectively, for samples collected 4–8 weeks after PCR testing. The SARS-CoV-2 RBD assay provided poorer classification with an AUC value of 0.883 for the same samples.

To assess the validity of our pre-established thresholds, we computed the optimal thresholds using data only from this study by identifying thresholds that maximize the sum of sensitivity and specificity. The pre-established thresholds and the optimal thresholds for this study are compared graphically in Fig. 3. For the spike and N IgG assays, the pre-established thresholds were close to optimal and no significant improvement in sensitivity or specificity could be achieved by adjusting the threshold. In contrast, the pre-determined threshold for the RBD IgG assay was lower than optimal and increasing the threshold from 0.244 AU/mL to 0.684 AU/mL greatly improved the specificity for samples collected 4 weeks or later after PCR testing from 64 to 87%, while causing a much smaller loss in sensitivity from 93 to 89%.

### Exploration of retest criteria

Although saliva collection is simple and intuitive, the potential for poor specimen quality should be addressed when specimens are self-collected without supervision and transported under uncontrolled conditions. We explored options for identifying specimens of high risk for providing inaccurate results including (i) the measurement of salivary antibodies that are expected to be universally abundant due to vaccinations or common natural infections, (ii) the measurement of total salivary immunoglobulin levels, and (iii) the measurement of background assay signals in the absence of an antigen target.

**Figure 4:**
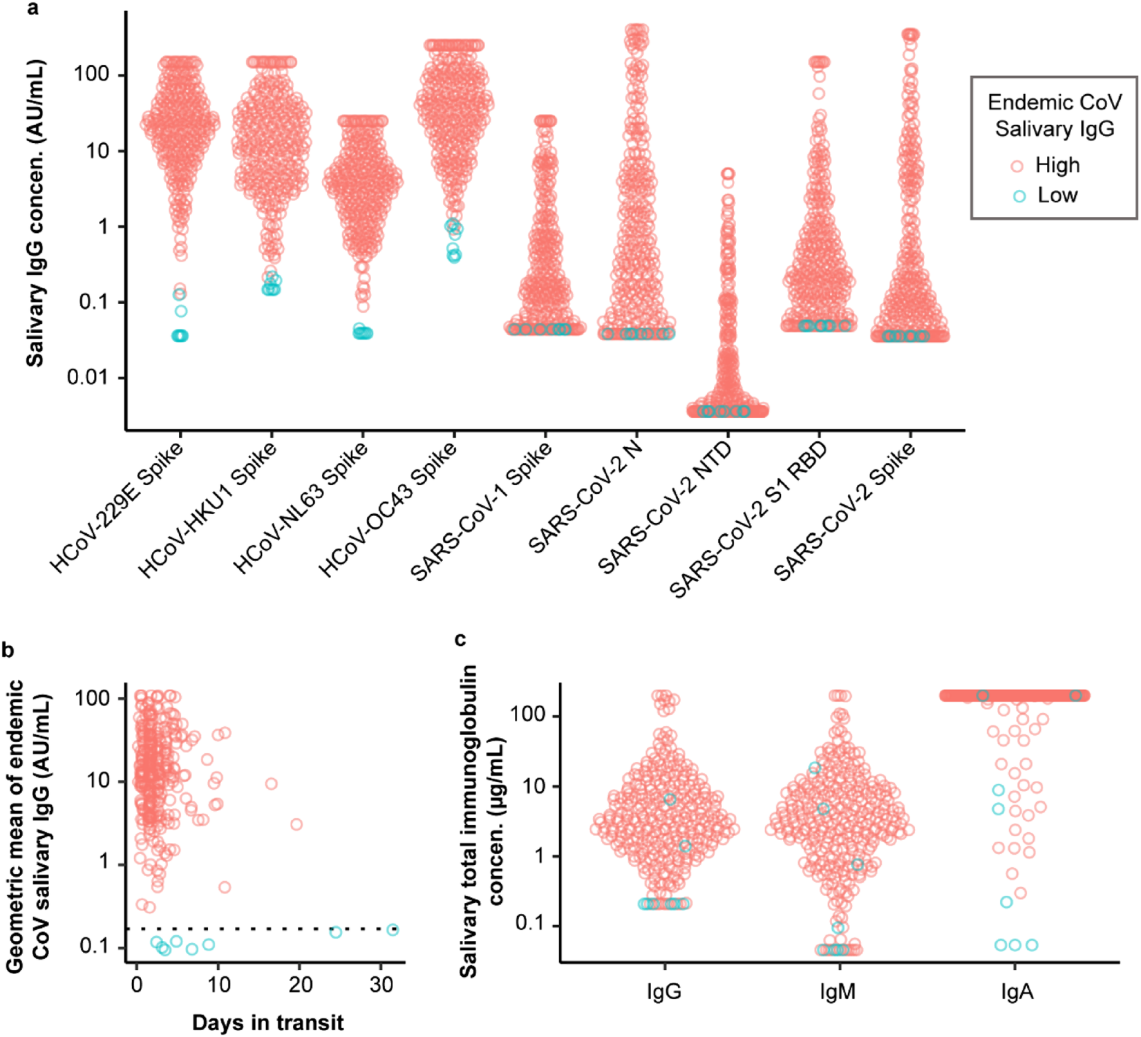
Concentrations of anti-spike and anti-N IgG measured in saliva of patients tested for SARS-CoV-2. Saliva was self-collected and returned via mail to a laboratory for analysis. For indirect serology, recombinant proteins representing the receptor binding domain (RBD) and N-terminal domain (NTD) of spike along with full-length spike and N antigen were multiplexed in wells of a 96-well plate. (**a**) IgG antibodies to four endemic coronaviruses were readily detected in nearly all samples. Eight samples (highlighted in blue) showed consistently low levels of antibodies to all coronaviruses. (**b**) 91% of samples were in transit for five days or less, and transit time was not strongly correlated with the mean level of salivary IgG for the four endemic coronaviruses (CoVs). Dotted line indicates geometric mean for the 5th percentiles of endemic CoV salivary IgG measured in a prior study of presumed naïve controls without prior diagnosis, household exposure, or symptoms of COVID-19^35^. (**c**) Total levels of immunoglobulins (IgG, IgM, and IgA) were also measured in saliva.

Prior infection with endemic coronaviruses is common^40,41^, so we expected that all donors would have high levels of antibodies to at least one of the four pre-COVID-19 endemic coronaviruses^42^. The multiplexed antigen panel used to measure antibodies to SARS-CoV-2 antigens also measured antibodies against the spike antigens for the four pre-COVID-19 endemic coronaviruses HKU1, NL63, OC43, and 229E (Fig. 4a). Nearly all specimens had readily detectable levels of antibodies to a spike protein of at least one endemic coronavirus. As an aggregate metric of reactivity to endemic coronaviruses, we computed the geometric mean of salivary IgG for HKU1, NL63, OC43, and 229E. We flagged eight outlier samples with geometric means below 0.17 AU/mL, which is the geometric mean of the 5th percentiles of the salivary IgG for these four antigens measured in a prior study^35^. These outliers appear to result from an issue with sample collection or sample deterioration, as opposed to the lack of immunity to an endemic coronavirus due to the absence of previous exposure or from general immunosuppression. In all cases where donors provided at least one other sample, normal levels of antibodies for endemic coronaviruses were measured at another time point. Sample deterioration due to delayed transit time in the mail could explain some of the flagged outliers, but not all of them. Two of the flagged samples were the samples with the longest transit times (> 20 days due to a general slowdown in mail during a period of this study), but the other 6 flagged samples were received within the target range of 5 days. For samples received within the target time range, there was no clear dependence of measured antibody levels with transit time (Fig. 4b). We note that for the 8 flagged samples, 6 were true negatives for SARS-CoV-2 infection by PCR testing, so excluding these samples did not significantly impact the reported sensitivity or specificity.

We also measured the total concentration of salivary IgG, IgM and IgA using a separate assay panel run at a different dilution (Fig. 4c). Median concentrations of total IgG, IgA and IgM were 3.3 µg/mL, ≥ 200 µg/mL (the top of the assay dynamic range at the selected sample dilution) and 3.4 µg/mL, respectively, which are comparable to the values we measured previously^35^ (1.8 µg/mL for IgG, 124 µg/mL for IgA and 3.7 µg/mL for IgM). Moreover, the IgG, IgA and IgM concentrations align with published ranges measured using a different assay and collection method (IgG range = 0.4–93 µg/mL^43^; IgA = 50.2 ± 19.1 µg/mL^44^; IgM = 0.5–13.0 µg/mL^45^). Low observed levels for the endemic coronaviruses were generally associated with low levels of total immunoglobulin. Of the eight samples that were flagged for low antibody levels against the four endemic coronaviruses, six samples had undetectable total IgG levels at the sample dilution used for the total immunoglobulin measurement, and four samples provided the lowest measured levels of total IgA (Fig. 4c).

Non-specific binding is another potential source of measurement error. Bovine serum albumin (BSA) was included as an antigen in the multiplex as a negative control. Specific binding of anti-BSA antibodies in samples should not occur due to the high concentration of BSA present in the assay diluents, therefore, binding to the non-BSA element in the antigen array should be indicative of antibodies that are able to bind non-specifically to the array surface. Non-specific binding, as assessed by signal for the control spot coated with BSA, was generally low (average of 191 counts). Two specimens from the same PCR negative donor were noted to exceed 5000 counts on the BSA coated spot, whereas a third intermediate sample from the same donor showed low non-specific binding.

## Discussion

We measured anti-SARS-CoV-2 antibodies in saliva self-collected at home by donors in the weeks following a nasal RT-PCR test. This is the first report to our knowledge of a “spit and mail” serology test for SARS-CoV-2. Importantly, we found that a diverse group of participants were able to self-collect saliva and send it for testing. Using only written instructions without in-person supervision or training, participants universally packaged their specimens according to UN3373 regulations. Specimens were of sufficient quality for analysis, and most participants provided multiple specimens. Multiplexing of antigens for SARS-CoV-2 alongside endemic coronaviruses identified approximately 3% of samples that appeared aberrant due to low levels of antibodies against the four pre-COVID-19 endemic coronaviruses, and those samples may require retesting due to poor sample quality. This study builds upon prior studies^18,19^ that found general acceptance for self-collected specimens by demonstrating feasibility of a “spit and mail” approach using a highly scalable kit.

Among participants whose PCR-tests were positive for SARS-CoV-2, the kinetics of changes in salivary antibodies closely paralleled the kinetics of serum antibodies. Antibodies appeared in saliva as early as the first week after PCR testing, and the concentration of salivary antibodies increased over the course of two months, which has also been reported for serum^46^. Our study indicates that measuring salivary antibodies can be an alternative to measurement using serum or finger-stick blood. Among PCR-positive participants who returned at least one sample 2–4 weeks after testing, 96% had detectable anti-SARS-CoV-2 spike IgG, which is notable since participants were recruited among patients with mild to moderate disease not requiring hospitalization. In a large study of individuals who recovered from SARS-CoV-2 infection, 91.1% (1,107 of 1,215) were seropositive based on serum testing using a different assay^47^. Testing among outpatients is more challenging since a systemic humoral response may be delayed or absent in some mild cases^48^, whereas seroconversion occurs in nearly 100% of hospitalized patients^46,49^. The sensitivity for detecting asymptomatic infection may be lower since individuals with mild cases tend to have less robust immune responses compared to individuals with severe disease^50–53^.

Samples were classified as antibody positive or negative based on thresholds determined in a previous study of self-collected saliva samples. Using these thresholds, we found that our assays for IgG against SARS-CoV-2 Spike and N antigens provided good sensitivity and specificity for classifying subjects based on COVID PCR test results. The ability to simultaneously measure IgG to both N and Spike proteins may potentially distinguish individuals who have been infected with SARS-CoV-2 (reactivity to both N antigen and Spike) from those that have been vaccinated without a prior infection (reactivity to Spike only). Poorer performance was observed for IgG to SARS-CoV-2 RBD. ROC analysis showed that the pre-determined threshold was not optimal. It is not clear why the optimal thresholds for the RBD IgG assay would be different for the earlier and current studies and why the difference would only affect one of the assays. The difference could be associated with differences in how self-collected samples were submitted for testing (collection in drop boxes vs. shipping by mail) or differences in the testing population (healthy individuals vs. individuals who presented for COVID testing and may have symptoms of a respiratory infection).

A limitation of this study is that we did not examine matched serum or finger-stick blood samples. Correlation of salivary antibody concentrations with finger-stick blood antibodies would help to determine the reason for the few cases when antibody levels were not as expected based on PCR testing. In particular, some participants may have previously been infected with SARS-CoV-2 prior to the nasal swab PCR test. Another limitation is that we did not measure reactivity of salivary IgA to SARS-CoV-2 antigens, which could potentially provide information that is different and complementary to IgG reactivity when assessing immune status.

Due to a lack of validated criteria to exclude samples based on atypical antibody testing, we excluded only two samples that were affected by prolonged postal delays. Measuring antibodies against endemic coronaviruses was identified as a potential approach, which can be easily multiplexed with the SARS-CoV-2 serology measurements, to flag specimens with suspected technical issues. Establishing retest criteria is recommended before the assay can be used for clinical testing. A geometric mean of antibody levels to four endemic coronaviruses is a promising metric for flagging suspect samples, especially because multiplex assays can obtain this metric without additional sample volume or processing. Additional studies with matched serum samples, or saliva samples collected under ideal conditions, are needed to refine and validate methods for identifying samples that may be affected by collection or handling issues.

Overall, we show the feasibility of a “spit and mail” test for large-scale serologic testing. The easy-to-use kit could be used by a diverse group of participants. The assays require minimal sample handling and can be performed rapidly using an automated analyzer capable of high-throughput testing. Potential uses include epidemiologic studies that require identifying people who have previously been infected or vaccinated. Also, saliva tests may find utility for monitoring durability of immunity.

## Methods and materials

### Participant recruitment and enrollment

Participants were recruited among adult patients tested for SARS-CoV-2 infection at Kaiser-Permanente clinics in Maryland via RT-PCR testing of nasal swabs. After the diagnostic test was performed, patients were invited to participate. All tested patients were eligible to participate regardless of symptoms, exposure risk, or reason for testing (e.g., pre-surgical evaluation, contact tracing). Participants who agreed to participate were provided with an enrollment box containing instructions, informed consent form, and three self-collection kits. All participants were recruited via a telephone call with the exception of one, who was recruited at a testing clinic and provided an enrollment box. Participants recruited via phone were shipped an enrollment box that arrived within 1–3 days after enrollment. Informed consent was obtained from all participants via a signed barcoded informed consent form and HIPAA authorization, which were mailed back to Kaiser-Permanente researchers. Participants were instructed to self-collect saliva on the day they received the kit and then at 10 and 30 days after their test. Saliva was returned directly to Meso Scale Diagnostics, LLC. (MSD) via the provided pre-paid mailers. MSD tracked participants through bar codes associated with the self-collection kits and had no access to identifiable personal information. Study procedures were approved by the IRB of Kaiser-Permanente. The KPMAS IRB reviewed, approved, and monitored the data collection, management, and analysis protocol in accordance with relevant guidelines/regulations.

### PCR-confirmed diagnosis of SARS-CoV-2 infection

Nasal swabs were tested by RT-PCR using a SARS-CoV-2 NAAT and a PAN-SARS Coronavirus NAAT. SARS-CoV-2 NAAT amplified the unique ORF1ab non-structural region, and the PAN-SARS Coronavirus NAAT amplified the conserved pan-Sarbecovirus structural protein envelope E gene. The results were interpreted as positive if either the ORF1ab alone or both the ORF1ab and E gene resulted in a positive result for the detection of SARS-CoV-2. Detection of the pan-Sarbecovirus E gene alone resulted in a presumptive positive result. Results were deemed negative if neither target was detected.. Results were obtained from the participant’s electronic medical record by Kaiser-Permanente (KP) researchers. All participants tested either positive or negative, with no participants testing positive for the pan-Sarbecovirus E gene but negative for the ORF1ab alone.

### Collection and transport of saliva

As described previously^35^, saliva was collected into a 2 mL screw-cap centrifuge tube (Sarstedt #72.609 with screw cap 65.716.xxx) using the Saliva Collection Aid (SCA; Salimetrics 5016.02), which is a straw-like device cleared by the FDA for collection of samples from adults and children. Centrifuge tubes were pre-labeled with barcodes that matched barcodes on informed consent forms in order to associate samples with participants in a deidentified manner. The screw cap contained an O-ring in order to be compliant with UN3373 Category B shipping requirements. Donors were instructed to wait 30 min after eating, drinking, or smoking before drooling into the tube according to the manufacturer’s directions. Donors were also instructed to provide saliva without assistance from others. The donors capped the tube, and placed it inside a biohazard bag (VWR 11215-684) containing an absorbent pad (ThermoSafe ZORB66). Samples were collected from July 2020 to March 2021 within the Washington, DC metropolitan area, prior to the broad availability of SARS-CoV-2 vaccines.

To return their samples, donors were instructed to place the biohazard bag containing the tube of saliva into a peel-and-seal cardboard mailer (Stephen Gould; MSD-CFM-SM) bearing a UN3373 label (LabelMaster L380B) and pre-paid shipping label as described previously^35^. The COVID-19 taskforce of the USPS reviewed the shipping materials and instructions for packing and mailing the specimens to ensure compliance with CDC guidelines and USPS regulations. A removable adhesive tag was attached to the back of each mailer alerting mail carriers to pick up the package.

### Saliva receipt and storage

Samples were returned anonymously to MSD via USPS First Class Mail. Upon delivery to MSD, saliva was frozen promptly at ≤ − 70 ºC without further processing. The date that saliva was mailed was documented as the date that the sample was picked up as determined by the USPS tracking history. For the initial samples, the accuracy of the USPS tracking record was cross-verified against the date entered by the patient on the informed consent form.

### Indirect serology

Indirect serology measurements were conducted using kits and reagents that are commercially available from MSD as described previously^35^. On the day of sample testing, saliva was thawed at room temperature. Saliva was centrifuged briefly to pull down any food particles or mucus. To assess sample quality, we visually verified that samples were saliva and not predominantly phlegm or mucus.

Prior to analysis, saliva samples were diluted five-fold by combining 20 µL of sample with 80 µL of a sample diluent (MSD® Diluent 2). Samples were assayed in a 96-well plate format using MSD V-PLEX® COVID-19 Coronavirus Panel 2 kits for measuring IgG (K15369U) antibody responses. Each well of the plates included an antigen array that enabled the multiplexed measurement of antibody responses against nine different coronavirus antigens as well as bovine serum albumin (BSA) as a negative control. These included four SARS-CoV-2 antigens (nucleocapsid protein, spike protein, spike receptor binding domain (RBD) and spike N-terminal domain (NTD)) and spike proteins from five other coronaviruses (SARS-CoV-1 and the four endemic coronaviruses 229E, HKU1, NL63, and OC43). Assay protocols were run according to the manufacturer’s recommended protocol for serum except for the use of sample diluents and dilution factors (as described above) that were optimized for saliva. Testing of saliva samples was carried out in an automated fashion using high-throughput automation developed at MSD. Time-to-result was approximately four hours.

For quantitation of antibody responses, an eight-point calibration curve was run in duplicate on all plates and the signals for each antigen were fit to a 1/Y^2^-weighted four parameter logistic (4PL) fit. Samples were run in duplicate and the antibody concentration against each antigen was calculated by back-fitting to the appropriate 4PL fit and correcting for dilution. The concentrations were presented in arbitrary units per mL (AU/mL) that were defined relative to the assigned values of the reference standard. Controls were also run in duplicate on each plate, including three serum-based controls (provided with the kit) and two saliva-based controls (pooled normal saliva sourced from Lee Biosolutions spiked with serum from COVID-19 patients).

### Measurement of total salivary antibodies

Total levels of IgG, IgM, and IgA immunoglobulin were measured using MSD’s Isotyping Panel 1 Human/NHP Kit (K15203D) according to the manufacturer’s directions as described previously^35^. Saliva was run at a dilution of 1,000-fold. Calibration and quantitation were carried out as described above for the indirect serology measurements.

### Data analysis

Data processing was performed in Excel. Graphing and statistical analysis were done in R. Concentrations below an assay’s limit of detection (LOD) were assigned concentration values equal to the LOD, which was calculated as the concentration corresponding to 2.5 standard deviations above the assay’s background signal. Concentrations exceeding the top calibrator were assigned the concentration of the top calibrator.

## Supplementary Information

Supplementary Information.

## Data Availability

The datasets generated and/or analyzed during the current study are available from the corresponding author on reasonable request.
