# Supplementary material for: Saliva-based SARS-CoV-2 serology using at-home collection kits returned via mail
Source: Sci Rep. 2022 Aug 18;12:14061. doi: 10.1038/s41598-022-17057-7 (PMC9387411; doi:10.1038/s41598-022-17057-7)
Supplement: Supplementary file 1 — Supplementary Information. [file 41598_2022_17057_MOESM1_ESM.docx]

Supplemental Materials for:

Saliva-based SARS-CoV-2 serology using at-home collection kits returned via mail

[Supplemental Table 1 – Accuracy of salivary serology assays at retrospectively classifying individuals as naïve or non-naïve to SARS-CoV-2 infection. 2](#_Toc90977607)

[Supplemental Figure 1 - Mailable kit for self-collection of saliva. 2](#_Toc90977608)

[Supplemental Table 2 – Range of immunoglobulin G (IgG) concentrations in saliva self-collected by donors and returned through the mail. 3](#_Toc90977609)

[Supplemental Table 3 – Range of total immunoglobulins in saliva self-collected by donors and returned through the mail. 4](#_Toc90977610)

[Supplemental Figure 2 – Salivary IgG for endemic coronaviruses in the weeks after a PCR test for SARS-CoV-2. 5](#_Toc90977611)

|  | <2 weeks after PCR test | | | 2-4 weeks after PCR test | | | 4-8 weeks after PCR test | | |
| --- | --- | --- | --- | --- | --- | --- | --- | --- | --- |
| Assay | **Area Under Curve (AUC)** | **AUC 95% CI** | **p-value** | **Area Under Curve (AUC)** | **AUC 95% CI** | **p-value** | **Area Under Curve (AUC)** | **AUC 95% CI** | **p-value** |
| SARS-CoV-2 Spike IgG | 0.761 | 0.66-0.87 | 1.2×10^-5^ | 0.970 | 0.95-0.99 | 6.9×10^-14^ | 0.926 | 0.85-1.0 | 9.1×10^-13^ |
| SARS-CoV-2 S1 RBD IgG | 0.697 | 0.57-0.82 | 0.001 | 0.894 | 0.84-0.95 | 4×10^-10^ | 0.883 | 0.79-0.98 | 1.6×10^-10^ |
| SARS-CoV-2 N IgG | 0.750 | 0.62-0.88 | 2.9×10^-5^ | 0.965 | 0.94-0.99 | 1.6×10^-13^ | 0.916 | 0.82-1.0 | 3.6×10^-12^ |

Supplemental Table 1 – Accuracy of salivary serology assays at retrospectively classifying individuals as naïve or non-naïve to SARS-CoV-2 infection. AUC values correspond to the graphs shown in Figure 3 of the main text. P-values computed using the Wilcoxon test.


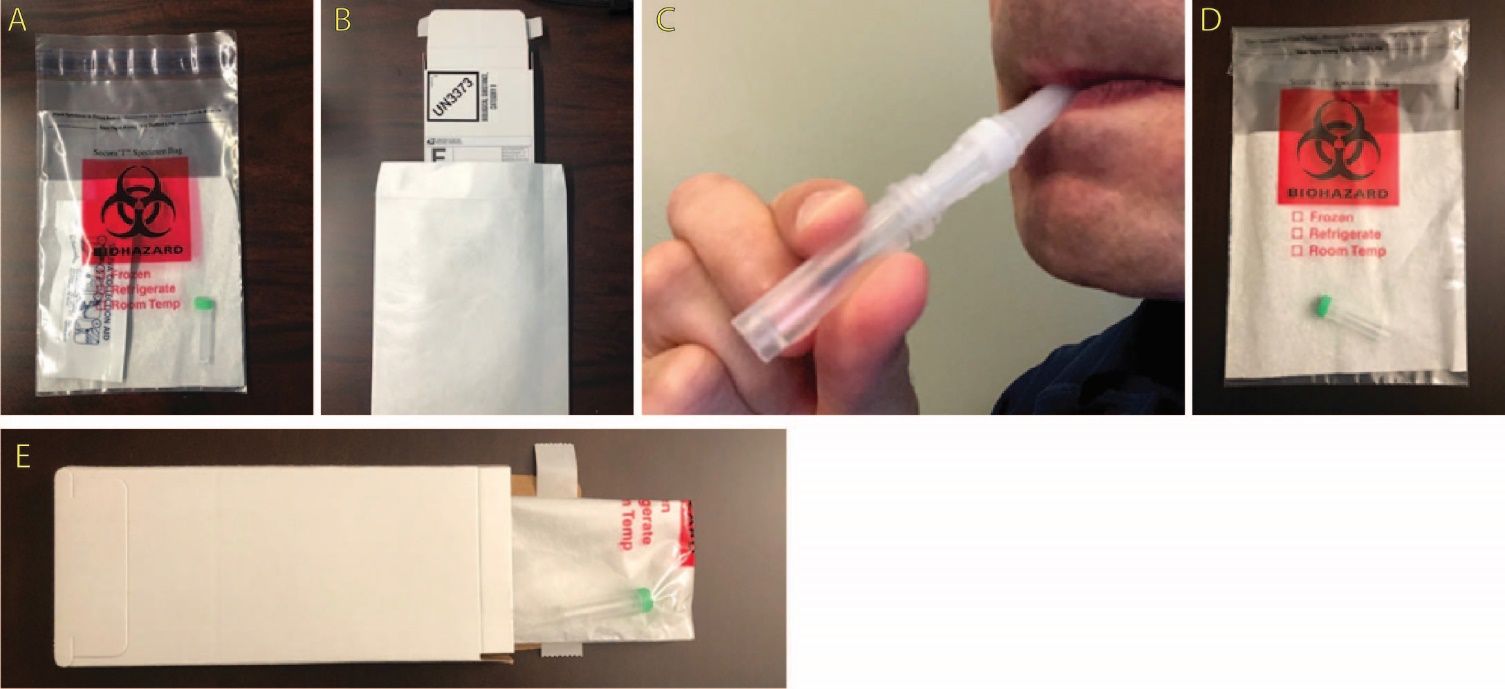


Supplemental Figure 1 - Mailable kit for self-collection of saliva. (A) The saliva collection kit consisted of a microcentrifuge tube, O-ring screwtop cap, and saliva collection aid (SCA) placed inside a sealable biohazard bag containing an absorbent material. The kit components were packaged in the same way as they were to be returned into the bag by the study participant. (B) The kit was placed inside of a peel-and-seal cardboard mailer displaying a UN3373 category B label. Saliva is collected into a microcentrifuge tube (C), which the participants enclose in a biohazard bag (D) and cardboard box (E) prior to mailing back to the laboratory

| Antigen and Isotype | Min | 5th percentile | 25th percentile | 75th percentile | 95th percentile | Max | Ratio of Max/Min | Ratio of 95%/5% | IQR | Median | Mean | Count |
| --- | --- | --- | --- | --- | --- | --- | --- | --- | --- | --- | --- | --- |
| HCoV-229E Spike IgG | 0.036 | 0.995 | 8.15 | 45.5 | 143 | 150 | 4.2e+03 | 140 | 37.3 | 21.4 | 36.2 | 302 |
| HCoV-HKU1 Spike IgG | 0.148 | 0.515 | 4.28 | 35.1 | 150 | 150 | 1e+03 | 290 | 30.8 | 12.1 | 29.4 | 302 |
| HCoV-NL63 Spike IgG | 0.0389 | 0.289 | 1.41 | 7.85 | 25 | 25 | 640 | 87 | 6.44 | 3.5 | 6.33 | 302 |
| HCoV-OC43 Spike IgG | 0.392 | 2.13 | 13 | 91.1 | 250 | 250 | 640 | 120 | 78.1 | 38.4 | 67.3 | 302 |
| SARS-CoV-1 Spike IgG | 0.0438 | 0.0438 | 0.0569 | 0.843 | 15.3 | 25 | 570 | 350 | 0.786 | 0.169 | 2.22 | 302 |
| SARS-CoV-2 N IgG | 0.0381 | 0.0381 | 0.0687 | 4.65 | 250 | 400 | 1e+04 | 6.6e+03 | 4.58 | 0.382 | 26.8 | 302 |
| SARS-CoV-2 NTD IgG | 0.00361 | 0.00361 | 0.00361 | 0.0352 | 0.865 | 5 | 1.4e+03 | 240 | 0.0316 | 0.00517 | 0.178 | 302 |
| SARS-CoV-2 S1 RBD IgG | 0.0488 | 0.0488 | 0.0969 | 0.928 | 18.9 | 150 | 3.1e+03 | 390 | 0.831 | 0.233 | 6.37 | 302 |
| SARS-CoV-2 Spike IgG | 0.0355 | 0.0355 | 0.0416 | 2.21 | 80 | 350 | 9.9e+03 | 2.3e+03 | 2.17 | 0.114 | 16.5 | 302 |

Supplemental Table 2 – Range of immunoglobulin G (IgG) concentrations in saliva self-collected by donors and returned through the mail. Sample concentrations are in AU/mL. Data shown for all 302 samples received from 121 unique participants.

| Isotype | Min | 5th percentile | 25th percentile | 75th percentile | 95th percentile | Max | Ratio of Max/Min | Ratio of 95%/5% | IQR | Median | Mean | Count |
| --- | --- | --- | --- | --- | --- | --- | --- | --- | --- | --- | --- | --- |
| IgA | 5.32e+04 | 7.22e+06 | 2e+08 | 2e+08 | 2e+08 | 2e+08 | 3.8e+03 | 28 | 0 | 2e+08 | 1.82e+08 | 302 |
| IgG | 2.07e+05 | 2.74e+05 | 1.35e+06 | 8.04e+06 | 3.57e+07 | 2e+08 | 970 | 130 | 6.68e+06 | 3.26e+06 | 1.13e+07 | 302 |
| IgM | 4.54e+04 | 6.23e+04 | 1.19e+06 | 8.6e+06 | 3.53e+07 | 2e+08 | 4.4e+03 | 570 | 7.4e+06 | 3.41e+06 | 1.06e+07 | 302 |

Supplemental Table 3 – Range of total immunoglobulins in saliva self-collected by donors and returned through the mail. Sample concentrations are in pg/mL. Data shown for all 302 samples received from 121 unique participants.


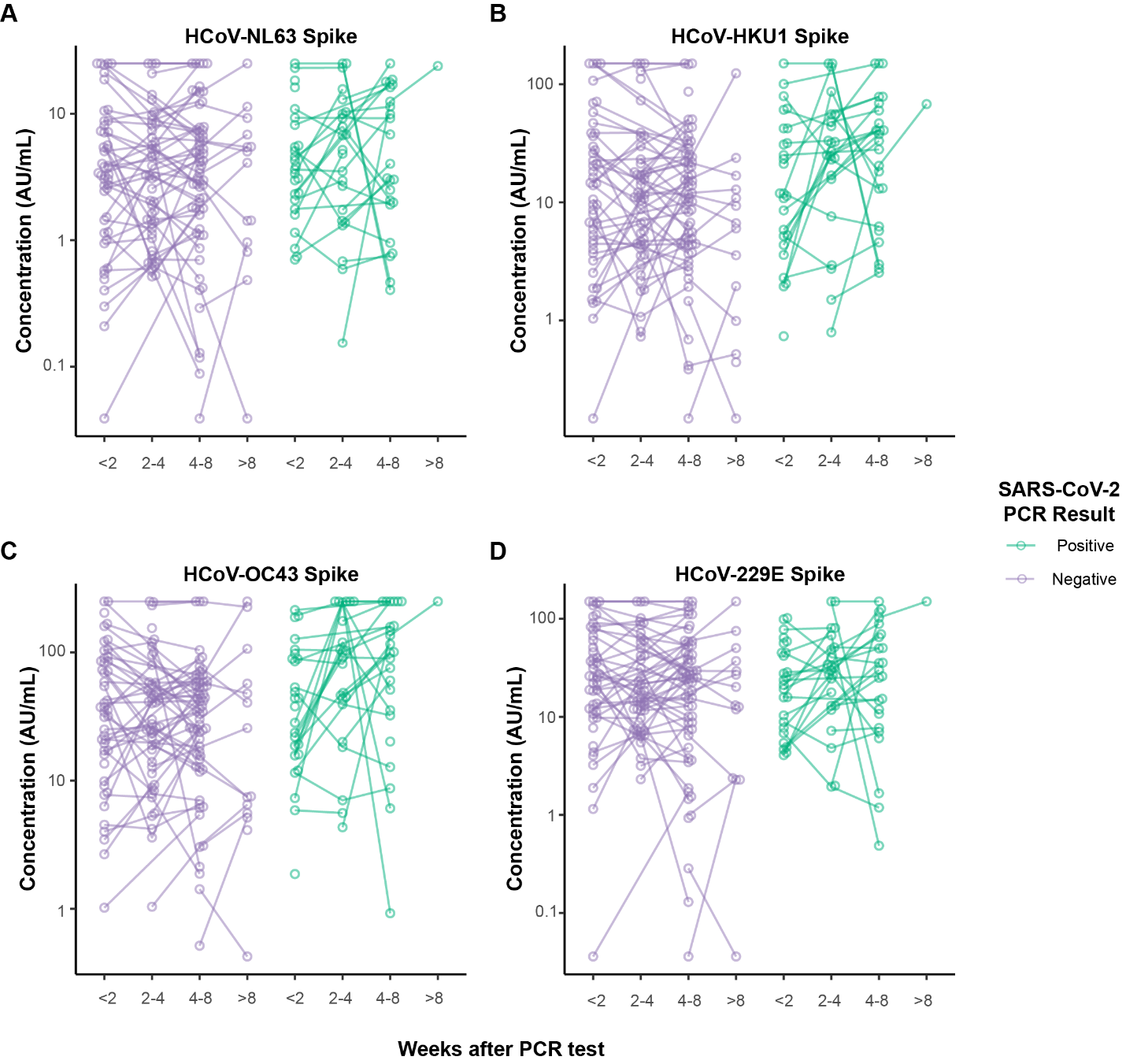


Supplemental Figure 2 – Salivary IgG for endemic coronaviruses in the weeks after a PCR test for SARS-CoV-2. IgG reactivity to the spike proteins for (a) NL63, (b) HKU1, (c) OC43, and (d) 229E was measured in saliva provided by the same 121 participants whose reactivity to SARS-CoV-2 antigens is shown in Figure 1 of the main manuscript. Participants provided up to three samples in the weeks after receiving a PCR test for SARS-CoV-2. Matched samples provided by the same donor are connected with lines. 81 participants tested negative for SARS-CoV-2 (colored purple), and 40 participants tested positive (colored green).
